# Shifting Our Perspective for the Future of Assessment and Intervention Science

**DOI:** 10.32872/cpe.6197

**Published:** 2021-03-10

**Authors:** Maria Karekla

**Affiliations:** a Department of Psychology, University of Cyprus, Nicosia, Cyprus

A big chunk of my early years in graduate school was spent learning about psychopathology and the diagnostic systems that categorize these. We learned about prevalence, contributing factors, how to assess and differentially diagnose individuals with psychopathological problems. When I started my clinical work, I was shocked to encounter that the reality of clinical practice was far from the information I learned in my psychopathology courses. Almost all clients, would not fit properly under one diagnosis, comorbidity was the norm, and I discovered that assigning a diagnosis was not particularly helpful for my case conceptualizations and choice of treatment. Since those days, even though I have seen hundreds of patients, I am still looking for the classic book example of a panic patient. As for depression, it is fascinating to me that I can give the same diagnosis to a patient who presents with loss of appetite, low energy, excessive sleepiness, and catatonic-like symptoms, as to a patient who presents with concentration difficulties, increased appetite, difficulty sleeping, and restlessness. How does our training in a topographical approach to psychological suffering with the search for syndromes (collection of signs and symptoms) prepare us for clinical practice and effective intervention? What are our diagnostic systems useful for? Interestingly, even the task force on DSM-5 (American Psychiatric Association, 2013) acknowledges the shortfall of this approach in “uncovering etiologies”, recommending intervention strategies, and have gone as far as to propose that a “paradigm shift may need to occur” (Kupfer, First, & Regier, 2002).

Beyond assessment and diagnosis, in the realm of treatment, psychological intervention training is driven by theories, traditions, or schools of thought (e.g., cognitive-behavioral, humanistic, psychodynamic). In training and education, we focus on teaching students’ tools, techniques, and approaches, almost like cookbooks, ignoring that the reality of practice or even cooking, is far from the strict following of a specific mechanistically applied set of tools. Inflexible and strict devotion to a particular approach has hindered scientifically based development of psychotherapy, has propagated bias and impeded progress and communication among therapists, and has prevented the investigation of common mechanisms that may drive therapeutic changes in individuals who suffer and seek services (Hofmann, 2020; Rief, 2021). Going back to the reality of human suffering, if we examine the World Health Organizations’ top 10 diseases causing the most deaths worldwide (WHO, 2020a, 2020b), we will notice that these include heart disease, stroke, chronic obstructive pulmonary disease, respiratory infections, neonatal conditions, lung-related cancers, Alzheimer’s and dementia, diarrheal diseases, diabetes, and kidney diseases. What is common among all these top killers? Common to all these are maladaptive health-related behaviors, dysfunctional coping, and behaving and all can be aided with the realm of the work we do as clinical psychologists- behavior change. Yet, despite important scientific advances, current treatments are hindered by these dysfunctional behaviors and clinicians' inability to help patients overcome them. Therefore, a change of perspective is needed on how we approach human suffering, and under what circumstances, how and where we intervene.

One such new perspective shift came from The National Institute of Health (NIMH, 2021) RDoC framework. This approach aimed to examine psychopathology as dysregulation of particular neurobiological and behavioral systems, including affective valence systems, cognitive systems, social systems, attachment processes, and arousal systems (Cuthbert, 2014). The goal is to translate progress in behavioral and neuroscience to improve understanding of psychopathology and develop new and tailored treatments. It remains to be seen whether this framework will prove helpful in remedying the problems posed above. Another recent development comes from Hofmann and Hayes (2019, p. 47), who are extending the question posed by Gordon Paul in 1969 and ask: “What core biopsychosocial processes should be targeted with this client given this goal, in this situation, and how can they most efficiently and effectively be changed?”. With this question and their new conceptual developments of a process-based approach couched within the umbrella of evolutionary science, they raise a different claim (see Hayes, Hofmann, & Ciarrochi, 2020). In this approach, assessment procedures and therapy can and should be linked via mechanisms of action implicated in the maintenance and treatment of suffering and the promotion of well-being.

Research from my laboratory and others around the world are presently attempting to establish necessary parameters so as to be able to result in directly linking mechanisms of action (change processes via which psychotherapeutic change can occur) with intervention choices and outcomes in an iterative, bottom-up manner. We recently proposed that a successful coupling of assessment and treatment depends on the basic core mechanisms of action identified and measured (Gloster & Karekla, 2020). Such candidate mechanisms need to: 1) be malleable and amenable to experimental manipulation, 2) demonstrate robustness across contexts, 3) be tested across time ideographically, and 4) be tested across multiple levels of analysis (e.g., biological, genetic, psychophysiological, and behavioral). Adopting such a multi-method, multi-level perspective in the exploration of mechanisms of action can move us towards functional process-based alternatives to approaching human suffering. When this is couched within a coherent theory such as that of evolutionary science (see Hayes, Hofmann, & Ciarrochi, 2020), we may be able to achieve meaningful progress towards our aim of better serving the humans who suffer and seek our services. I hope that as a field we will shift our perspective to a more functional, contextualistic, and process-based approach for the future of our assessment and intervention science.
